# Fumarate hydratase loss promotes mitotic entry in the presence of DNA damage after ionising radiation

**DOI:** 10.1038/s41419-018-0912-3

**Published:** 2018-09-06

**Authors:** Timothy I. Johnson, Ana S. H. Costa, Ashley N. Ferguson, Christian Frezza

**Affiliations:** Medical Research Council Cancer Unit, University of Cambridge, Hutchison/MRC research centre, Box 197, Cambridge Biomedical Campus, Cambridge, CB2 0XZ United Kingdom

## Abstract

An altered response to DNA damage is commonly associated with genomic instability, a hallmark of cancer. Fumarate hydratase (FH) was recently characterised as a DNA repair factor required in non-homologous end-joining (NHEJ) through the local production of fumarate. Inactivating germline mutations in FH cause hereditary leiomyomatosis and renal cell cancer (HLRCC), a cancer syndrome characterised by accumulation of fumarate. Recent data indicate that, in FH-deficient cells, fumarate suppresses homologous recombination DNA repair upon DNA double-strand breaks, compromising genome integrity. Here, we show that FH loss confers resistance to DNA damage caused by ionising radiation (IR), and promotes early mitotic entry after IR in a fumarate-specific manner, even in the presence of unrepaired damage, by suppressing checkpoint maintenance. We also showed that higher levels of DNA damage foci are detectable in untreated FH-deficient cells. Overall, these data indicate that FH loss and fumarate accumulation lead to a weakened G2 checkpoint that predisposes to endogenous DNA damage and confers resistance to IR.

## Introduction

Fumarate hydratase (FH) is a nuclear-encoded metabolic enzyme that catalyses the reversible conversion of fumarate to malate in the mitochondria and cytosol. Within the mitochondria, FH participates in the tricarboxylic acid (TCA) cycle, whereas in the cytosol it buffers fumarate produced from cytosolic reactions such as during purine biosynthesis and from the production of arginine from argininosuccinate in the urea cycle. FH loss leads to hereditary leiomyomatosis and renal cell cancer (HLRCC), a cancer syndrome characterised by benign smooth muscle tumours in the skin and uterus, and type II papillary renal cancer^1^. Genetic analysis revealed that while patients inherit one mutated allele, tumour formation is due to the loss of the remaining wild-type FH allele (loss of heterozygosity, LOH), identifying FH as a bona fide tumour suppressor^1^. HLRCC is characterised by the aberrant accumulation of FH’s substrate, fumarate, which has been implicated in tumorigenesis and recently defined an “oncometabolite”. Among different functions, fumarate was shown to inhibit various αKG-dependent dioxygenases, such as the hypoxia inducible factor (HIF) prolyl hydroxylases^2^ and histone and DNA demethylases^3,4^ leading to profound epigenetic changes associated with tumour formation.

Defects in DNA damage repair and genome instability have long been associated with tumorigenesis, as is emphasised by the numerous hereditary disorders, such as Xeroderma Pigmentosum and Fanconi anaemia, that predispose to cancer due to mutations in DNA repair genes^5^. DNA double-strand breaks (DSBs) are considered the most toxic DNA lesion and cells rely on multiple repair pathways for their resolution, though many of the proteins in these pathways are commonly mutated in cancer. There are two main pathways responsible for the repair of DSBs, non-homologous end-joining (NHEJ) and homologous recombination repair (HRR). NHEJ operates throughout the cell cycle, whereas HRR can only be used when a homologous sequence is available after replication in S and G2 phase. Since HRR uses a homologous sequence as a template, it is generally considered less error-prone than NHEJ, although the exact nature of the DSB is a major factor in choice between these two pathways^6^. The major function of these repair pathways is to resolve DNA damage that, if left unrepaired, could compromise the genomic integrity of the cell and its future progeny during cell division. In order to facilitate repair, cells have developed cell cycle checkpoints to halt or slow the cell cycle in response to DNA damage. Yet, even a low number of DNA lesions allowed to persist into mitosis could result in genomic re-arrangements, further genomic instability and cancer initiation^7^.

FH has emerged as an important player in regulating the response to DNA damage. It was found that yeast cells lacking cytosolic FH are more sensitive to inducers of DSBs, including ionising radiation (IR) and hydroxyurea^8^. These findings identified a “moonlighting” role for FH in the nucleus after DNA damage and provided the first evidence that FH is a component of the DNA damage response (DDR). More recent evidence links FH nuclear activity and NHEJ where FH is phosphorylated by the catalytic subunit of DNA-PK (DNA-PKcs) upon induction of DSBs, allowing FH to bind to histone variant H2A.Z^9^. Reverse activity of FH, the conversion of malate to fumarate, was shown to lead to a local accumulation of fumarate that is hypothesised to inhibit an alpha-ketoglutarate (αKG)-dependent lysine demethylase, KDM2B. The resulting persistence of di-methylated Histone H3 at lysine 26 facilitates the binding of pro-NHEJ proteins. Furthermore, it was recently shown that elevated levels of fumarate correlate with increased endogenous damage, lower HRR efficiency and increased sensitivity to poly-ADP ribose polymerase (PARP) inhibitors in vivo^10^. However, the effects of fumarate on cell cycle progression upon IR is unknown.

In this work, we investigated the response to DNA damage in the human FH-deficient cell line UOK262 versus its wild-type FH overexpressed counterpart, UOK262pFH, and reveal that FH loss leads to resistance to IR and promotes early mitotic entry after IR even in the presence of unrepaired damage in a fumarate-specific manner. Interestingly, pro-NHEJ factors are more readily recruited to sites of damage in FH-deficient cells and higher levels of DNA damage foci are detectable in untreated cells and mitotic chromosomes. This work provides evidence that fumarate accumulation, in the context of FH deficiency, promotes genomic instability by inducing endogenous damage that bypasses a weakened G2 checkpoint.

## Results

### FH-deficient cells exhibit reduced sensitivity to IR

**Fig. 1:**
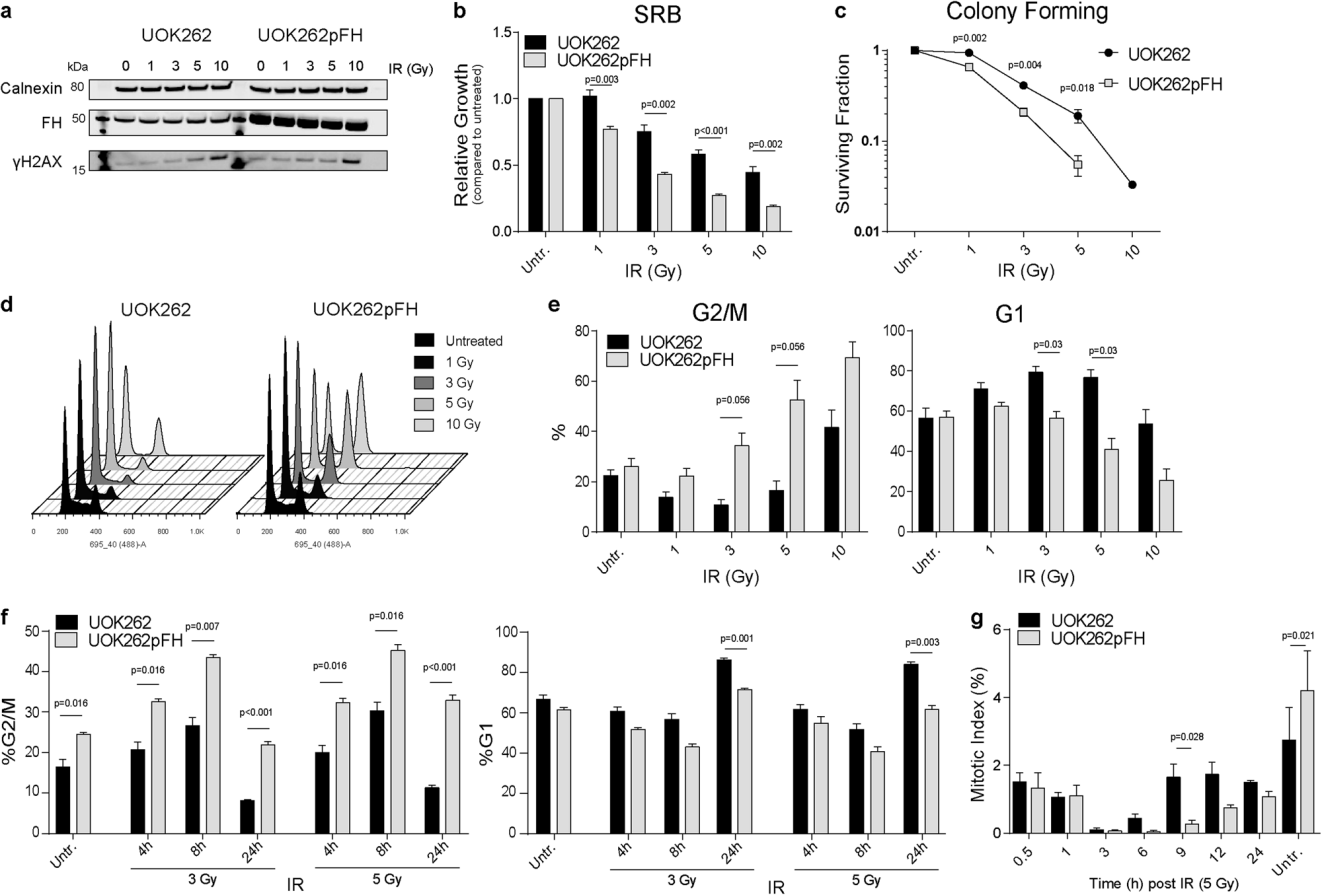
FH-deficient UOK262 cells exhibit resistance to ionising radiation (IR) and distinct cell cycle changes after IR. **a** Western blot analysis of protein extracts harvested 1 h after the respective IR doses using anti-FH and anti-phospho-Serine 139 H2AX (γH2AX) antibodies with anti-calnexin antibodies used as a loading control. **b**, **c** Relative growth as compared to untreated control using **b** sulforhodamine B (SRB) absorbance at 564 nm or **c** colony-forming assays after increasing IR doses. **d**, **e** Representative plot of DNA content (**d**) and quantification of cell cycle phases (**e**) determined using propidium iodide (PI) at increasing doses of IR. **f** Quantification of the G2/M and G1 population using PI at 4, 8 and 24 h after 3 or 5 Grays (Gy) of IR. **g** Mitotic index (MI) calculated at each time point based on number of pH3-positive cells as a percentage of total cells. All adjusted *p*-values calculated using multiple unpaired *t*-tests using the Holm-Sidak multiple comparison correction

To investigate the impact of FH loss on the DDR, we subjected previously characterised FH mutant and wtFH-expressing isogenic cell lines^4,11^ (hereafter denoted UOK262 and UOK262pFH, S1a) to increasing doses of IR and determined the impact on cell proliferation by sulforhodamine B (SRB) and colony-forming assays. Accumulation of fumarate, mitochondrial dysfunction and the rescue of these phenotypes with FH expression were confirmed using liquid chromatography–mass spectrometry (LC–MS) and measurement of oxygen consumption rate (OCR) respectively (Supplementary Fig. S1). Western blot analysis demonstrated the dose-dependent increase in phosphorylated Histone H2AX (γH2AX), a marker of DNA damage, 1 h after irradiation confirming the induction of DNA damage by IR (Fig. 1a). FH-deficient cells exhibit increased survival at multiple doses of IR, in both short-term (SRB) and long-term (colony-forming) proliferation assays compared to UOK262pFH cells (Fig. 1b, c). In particular, UOK262 cells exhibit enhanced long-term proliferation capacity even at high doses (5 and 10 Gy) of irradiation, suggesting these cells are able to maintain cell cycle progression after high levels of DNA damage. Since the relationship between induction of DSBs and cell cycle arrest is firmly established, we investigated whether the difference in survival is due to changes in cell cycle regulation after damage. We analysed DNA content by propidium iodide staining 24 h after irradiation and identified substantial changes in cell cycle phases after induction of DNA damage (Fig. 1d, e). In particular, a drop in the S phase population is evident in both cell lines, suggesting that entry into S phase is inhibited. Interestingly, UOK262pFH cells exhibit a more pronounced, dose-dependent G2/M arrest compared to UOK262 cells, which have a higher proportion of G1 cells (Fig. 1e). These results suggested that FH deficiency causes cells to either arrest in G1 or progress more rapidly through mitosis after damage. To distinguish between these two possibilities, we performed a time-resolved cell cycle analysis following 3 and 5 Gy IR. We found that the G2/M population increases at early time points in both cell lines compared to untreated cells (Fig. 1f). However, whereas the G2/M population significantly decreases after 24 h in UOK262 cells treated with both 3 and 5 Gy IR, the G2/M population in UOK262pFH cells remains higher (Fig. 1f). Overall, these results indicate that FH-deficient cells exhibit a faster mitotic progression that contributes to the diminished G2/M accumulation upon IR. To corroborate these results, we measured the mitotic marker, phospho-Histone H3, in response to IR (Supplementary Fig. S2a for a schematic). Mitotic index (MI) was significantly increased at 9 h post-IR in UOK262 cells (Fig. 1g), suggesting that these cells do indeed exhibit faster mitotic entry after IR.

### G2 arrest is not due to defective DNA repair

**Fig. 2:**
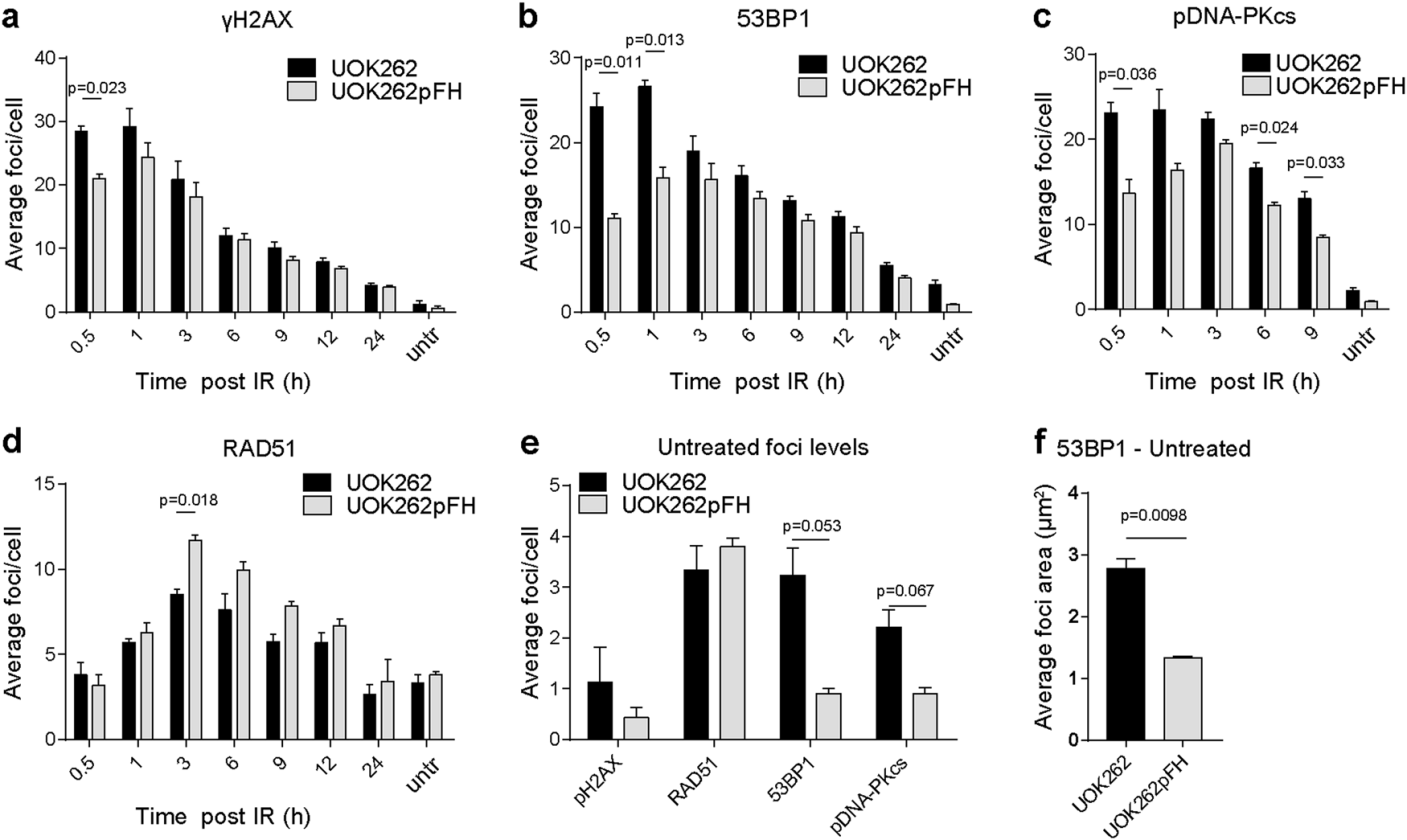
UOK262 and UOK262pFH cells efficiently repair double-strand breaks (DSBs). **a**–**d** Phospho-Serine 139 H2AX (γH2AX), 53BP1, phospho-Serine 2056 DNA-PKcs (pDNA-PKcs) and RAD51 foci count. **e** Foci levels of untreated cells re-plotted as a single bar graph. **f** Average 53BP1 foci area in untreated UOK262 and UOK262pFH cells. All adjusted *p*-values calculated using multiple unpaired *t*-tests using the Holm-Sidak multiple comparison correction

We then investigated the possible mechanisms underpinning the faster mitotic progression of FH-deficient cells upon IR. We first determined whether FH loss could affect the rate of DNA repair, a key factor for cell cycle progression. To this aim, we measured γH2AX, 53BP1, RAD51 and phosphorylated DNA-PKcs (S2056), key markers of total DNA damage, DSBs, HRR, and NHEJ, respectively. We irradiated cells with 5 Gy IR and assessed the kinetics and the abundance of these proteins using a high content microscope (representative images of DNA and foci masks shown in Supplementary Fig. S2b). UOK262 cells exhibit a higher level of γH2AX and 53BP1 foci at 0.5 and 1 h post-IR (Fig. 2a, b). Yet, all the DNA damage markers showed a time-dependent decrease in foci number (Fig. 2a–d), suggesting that the kinetic of DNA repair is similar in the two cell lines. Interestingly, UOK262 cells exhibit higher levels of phospho-DNA-PKcs foci (Fig. 2c) and lower levels of RAD51 foci (Fig. 2d). Furthermore, comparison of foci levels in untreated cells demonstrated that UOK262 cells have significantly higher levels of 53BP1 and phospho-DNA-PKcs foci suggesting an increase in endogenous damage (Fig. 2e). Indeed, under-replicated regions of DNA that persist into mitosis are marked by large 53BP1 foci in the subsequent G1 phase. Consistent with this, average 53BP1 foci area was significantly increased in untreated UOK262 cells (Fig. 2f). These results indicate that the early response to damage is heightened in FH-deficient cells, these cells exhibit increased endogenous damage, and that NHEJ is more predominant, in agreement with previous reports highlighting fumarate-dependent promotion of NHEJ^9^.

### FH-deficient cells display early mitotic entry

**Fig. 3:**
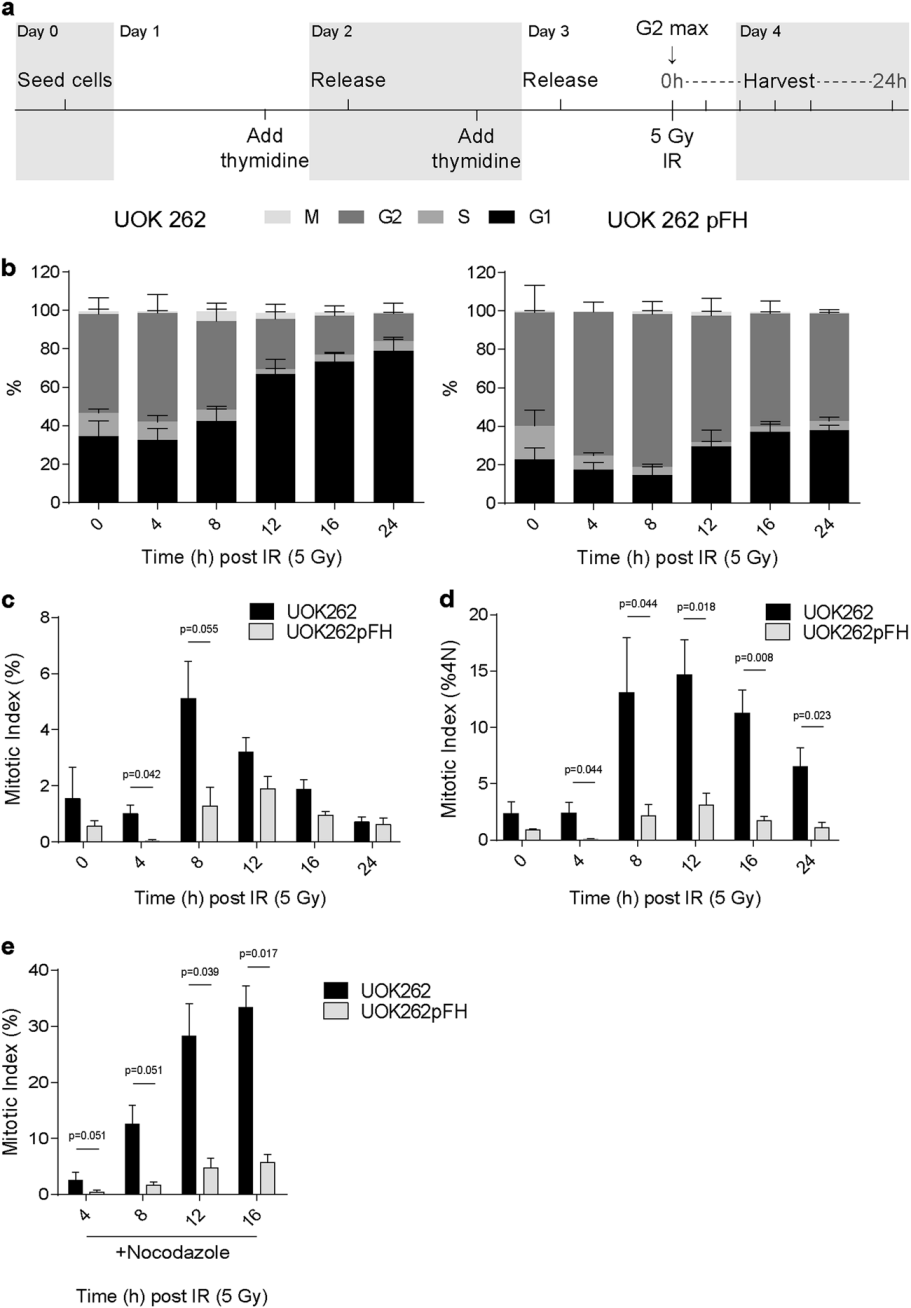
Synchronised UOK262 cells exhibit faster mitotic entry after IR. **a** Experimental outline detailing timings of thymidine synchronisation and irradiation/sample collection. **b** Representative graph of cell cycle populations (%) after IR treatment at maximal G2 content (0 h). **c**, **d** Number of phospho-Serine/Threonine-Proline MPM2 (p-MPM2)-positive cells was used for calculation of mitotic index (MI, **c**) and MI of 4N cells (**d**) after thymidine synchronisation and IR treatment. **e** Addition of the microtubule inhibitor, nocodazole, immediately after irradiation leads to accumulation of p-MPM2-positive mitotic cells. All adjusted *p*-values calculated using multiple unpaired *t*-tests using the Holm-Sidak multiple comparison correction

Since the rate of DNA repair could not explain the difference in MI upon DNA damage between the two cell types, we investigated the rate of the G2-M transition after IR. To this end, we used a double thymidine block protocol to synchronise S phase entry and maximise the G2 population (G2_max_) prior to irradiation (Fig. 3a and Supplementary Fig. S3). We irradiated cells at G2_max_ and time points were taken over 24 h before analysis using flow cytometry. Using DNA content and a marker of mitotic cells, phospho-MPM2 (p-MPM2), we found that UOK262 cells exhibit a significant increase in the mitotic population at 8 h after IR and an increase in G2 to G1 progression following 5 Gy IR (Fig. 3b, c). To take in to account differences in G2_max_ percentage, we also expressed MI as a percentage of 4N cells (Fig. 3d). These data demonstrate that mitotic progression is significantly higher in UOK262 across all time points from 8 h post-IR. Furthermore, when using the microtubule polymerisation inhibitor nocodazole to trap cells in mitosis, we found that over 30% of all cells were p-MPM2 positive in UOK262 cells by 24 h post-IR compared to <10% in UOK262pFH cells (Fig. 3e), suggesting that using nocodazole is effective in determining the net transition into mitosis over time. These data confirm that FH loss promotes G2-M transition after IR without affecting the kinetics of repair.

**Fig. 4:**
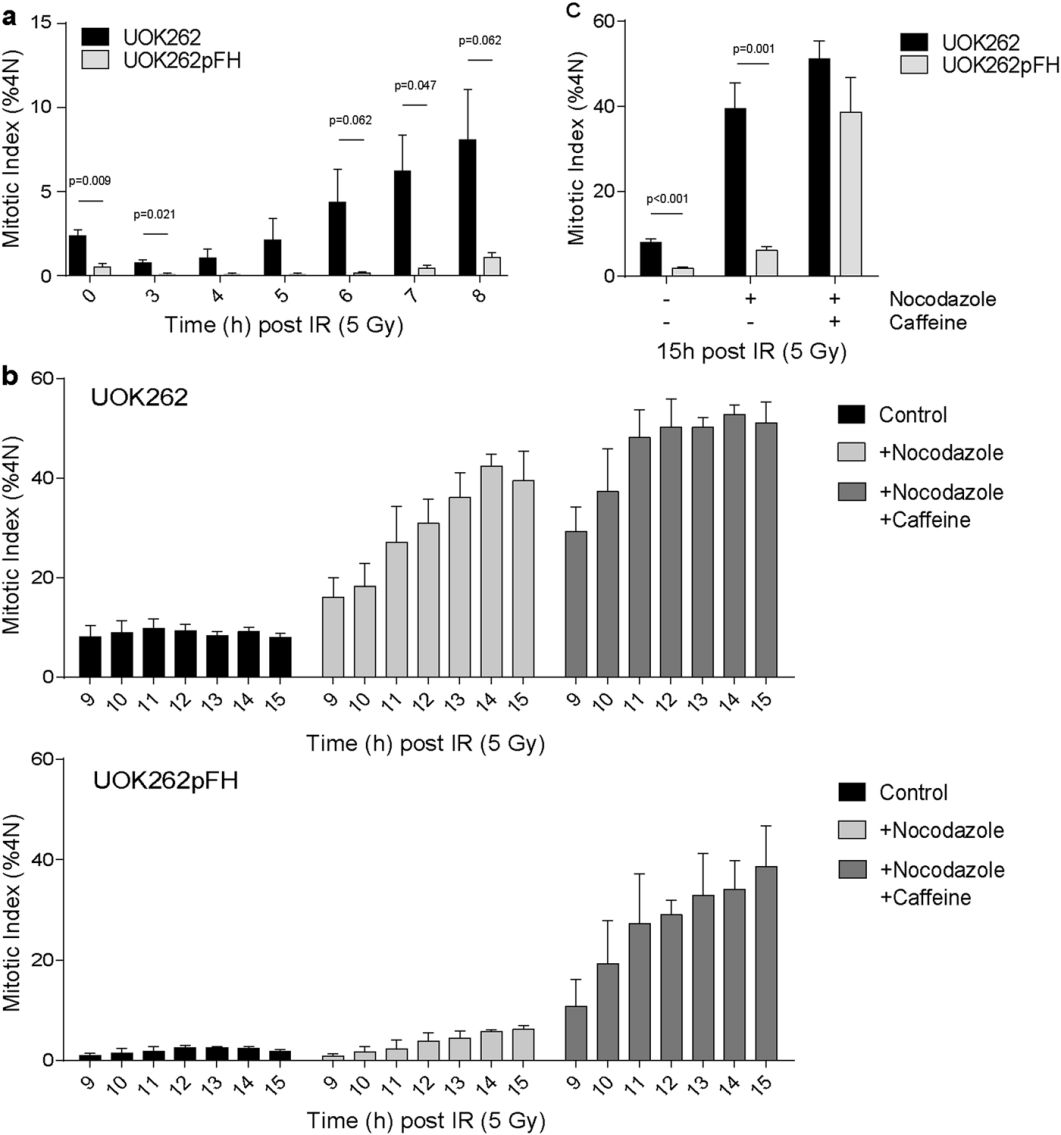
Caffeine treatment rescues G2 arrest in UOK262pFH cells. **a** Mitotic index (MI) of 4N cells (%4N) at early time points following 5 Gy IR. **b** MI (%4N) at later time points following 5 Gy IR including treatment with control, nocodazole alone or nocodazole in combination with caffeine. **c** Comparison of the final 15 h time point across all conditions, taken from **b** All adjusted *p*-values calculated using multiple unpaired *t*-tests using the Holm-Sidak multiple comparison correction

To assess the activity of the G2 checkpoint, we incubated thymidine-synchronised UOK262 and UOK262pFH cells with nocodazole in the presence or absence of caffeine, a known checkpoint inhibitor. At early time points, before the addition of caffeine, UOK262 cells exhibit a faster increase in 4N MI consistent with earlier data (Fig. 4a). Although UOK262 cells treated with caffeine and nocodazole accumulate in mitosis faster than those treated with nocodazole alone, the maximum level of mitotic cells is similar in both conditions (Fig. 4b, c). In contrast, mitotic accumulation in UOK262pFH cells in the presence of nocodazole is significantly increased by caffeine (Fig. 4b, c). These data identify a dysregulation of G2 checkpoint signalling as the major determinant of mitotic progression after IR in these cells.

### Fumarate accumulation is responsible for early mitotic entry upon IR

Since the loss of FH induces two major metabolic phenotypes, loss of mitochondrial function and accumulation of fumarate, we wanted to interrogate which metabolic alteration is responsible for our enhanced mitotic progression. To this end, we generated a human FH vector that lacks the mitochondrial targeting sequence and should localise only to the cytoplasm, hereafter called cytoFH. In addition, the V5 tag was substituted with green fluorescent protein (GFP) to allow for direct visualisation and cell sorting. After we transfected the modified FH vector into UOK262 cells under antibiotic selection, we sorted the resulting cells based on GFP fluorescence to obtain a polyclonal, GFP-positive cell line, UOK262cytoFH. Expression of the FH-GFP fusion protein was verified by western blot where the UOK262cytoFH cell line exhibited a band detected by an anti-FH antibody that displayed the expected molecular weight shift (Supplementary Fig. S4a). Cytoplasmic localisation of the fusion protein was verified by immunofluorescence analysis showing pan-cytoplasmic GFP signal as well as a staining pattern distinct from the mitochondrial-specific staining of tetramethyl rhodamine methyl ester (TMRM, Supplementary Fig. S4b) indicating the lack of mitochondrial import. More importantly, this cell line exhibits significantly reduced intracellular fumarate but maintained succinate accumulation, albeit partially, as well as an impairment in respiration (Supplementary Fig. S4c, d). This suggested that although the fumarate accumulation was reversed, the mitochondria remained dysfunctional, allowing us to differentiate between these two phenotypes in the context of DNA damage.

**Fig. 5:**
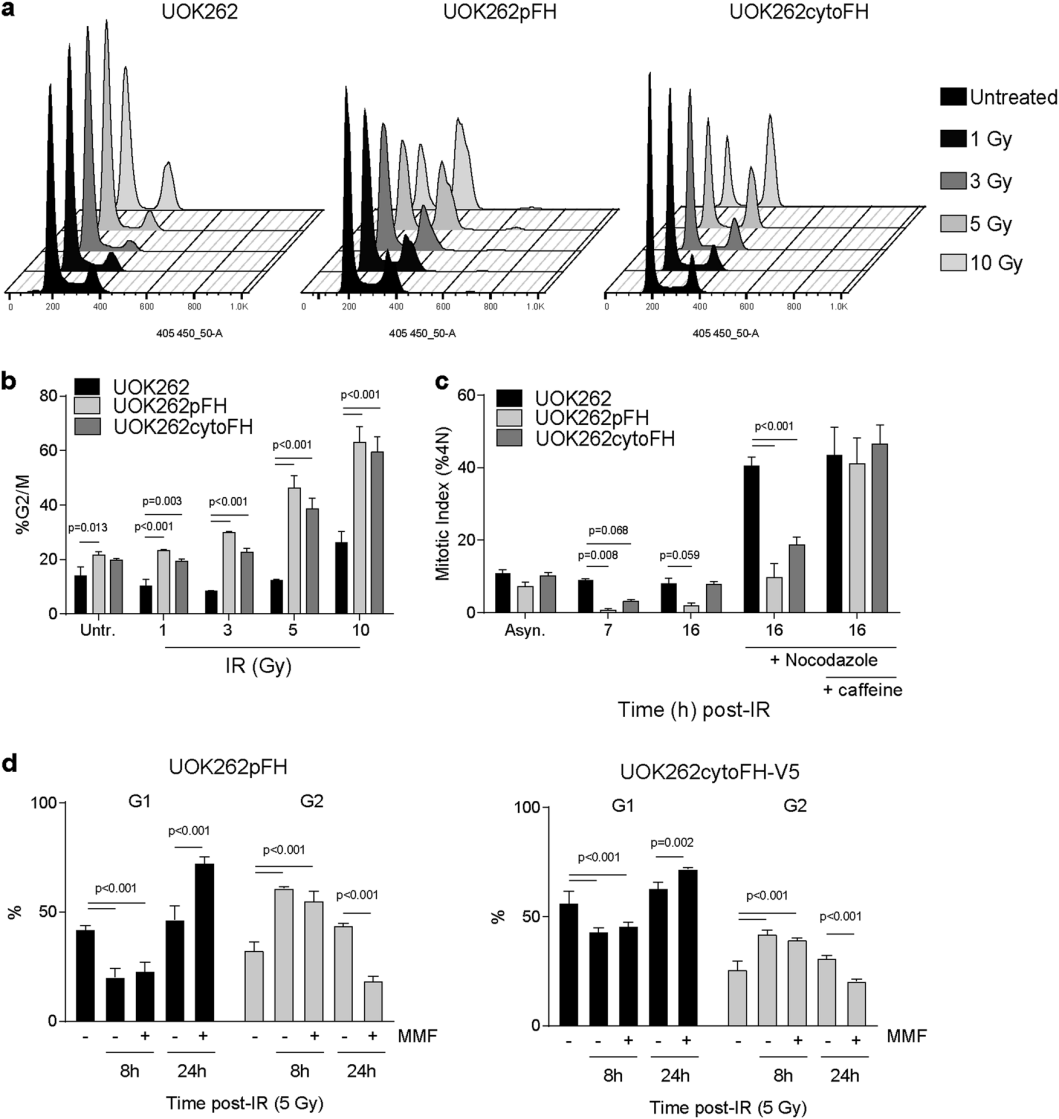
Fumarate directly impacts G2-M transition after IR. **a** Representative DNA content profiles after flow cytometric analysis of UOK262, UOK262pFH and UOK262cytoFH-GFP cells 24 h after increasing doses of IR. **b** Cell cycle phases were calculated using DNA content and percentage of 4N cells (G2/M) was plotted for each condition. **c** Mitotic index (MI, %4N) at 7 and 16 h following 5 Gy IR. Nocodazole with or without caffeine was added to the cells 7 h post-IR. **d** Quantification of G1 and G2 populations using DNA content and p-MPM2 at 8 and 24 h post 5 Gy IR with and without the cell-permeable fumarate derivative, monomethyl-fumarate (MMF) on the indicated cell lines. All adjusted *p*-values calculated using Tukey’s multiple comparison test

First, we assessed the sensitivity of UOK262cytoFH cells to increasing doses of IR by SRB and found they displayed a similar sensitivity profile to wtFH cells (Supplementary Fig. S4e), suggesting fumarate accumulation is responsible for the increased survival in UOK262 cells. Based on the correlation between IR sensitivity and the G2 arrest observed in UOK262pFH cells, we then analysed the cell cycle dynamics in asynchronous UOK262cytoFH cells by flow cytometry after incremental IR treatments. We found these cells also exhibited a sustained G2 arrest 24 h after IR in a dose-dependent manner, phenocopying wtFH expression (Fig. 5a, b). To interrogate the G2-M transition more specifically, we optimised cell cycle synchronisation of UOK262cytoFH cells by a double thymidine block (Supplementary Fig. S5) and irradiated cells at G2_max_. Like UOK262pFH cells, these cells fail to accumulate significantly in mitosis after IR in the presence of nocodazole suggesting that the G2-M transition is similarly impaired in these cells (Fig. 5c). However, these cells do exhibit partially increased MI at 16 h post-IR as compared to wtFH cells indicating a partial phenotypic rescue. This G2 arrest was rescued upon caffeine treatment (Fig. 5c), confirming the role of active checkpoint signalling in preventing mitotic entry in both UOK262pFH and UOK262cytoFH cells. Importantly, use of the specific Checkpoint kinase 1/2 (Chk1/2) inhibitor, AZD7762, instead of caffeine gave similar rescue of the G2 arrest observed in UOK262pFH and UOK262cytoFH cells, further supporting the role of checkpoint signalling in these cells (Supplementary Fig. S6).

Finally, to assess whether fumarate directly affects the G2/M checkpoint, we incubated cells with a cell-permeable form of fumarate, monomethyl-fumarate (MMF) and determined its effects on cell cycle upon IR. We found that the addition of MMF to FH- and cytoFH-reconstituted UOK262 cells immediately prior to IR lead to a decrease in G2 and corresponding increase in G1 24 h post-IR (Fig. 5d), suggesting fumarate has a direct role in G2-M transition. Importantly, the G2 population after 8 h post-IR was similarly elevated in both dimethyl sulfoxide (DMSO) and MMF conditions, indicating that the addition of MMF did not alter activation of the initial G2 checkpoint response. These data suggest that accumulation of fumarate, rather than mitochondrial dysfunction, leads to the dysregulation of the G2 checkpoint, earlier mitotic entry and increased survival after IR.

### FH-deficient cells enter mitosis even in the presence of DNA damage

**Fig. 6:**
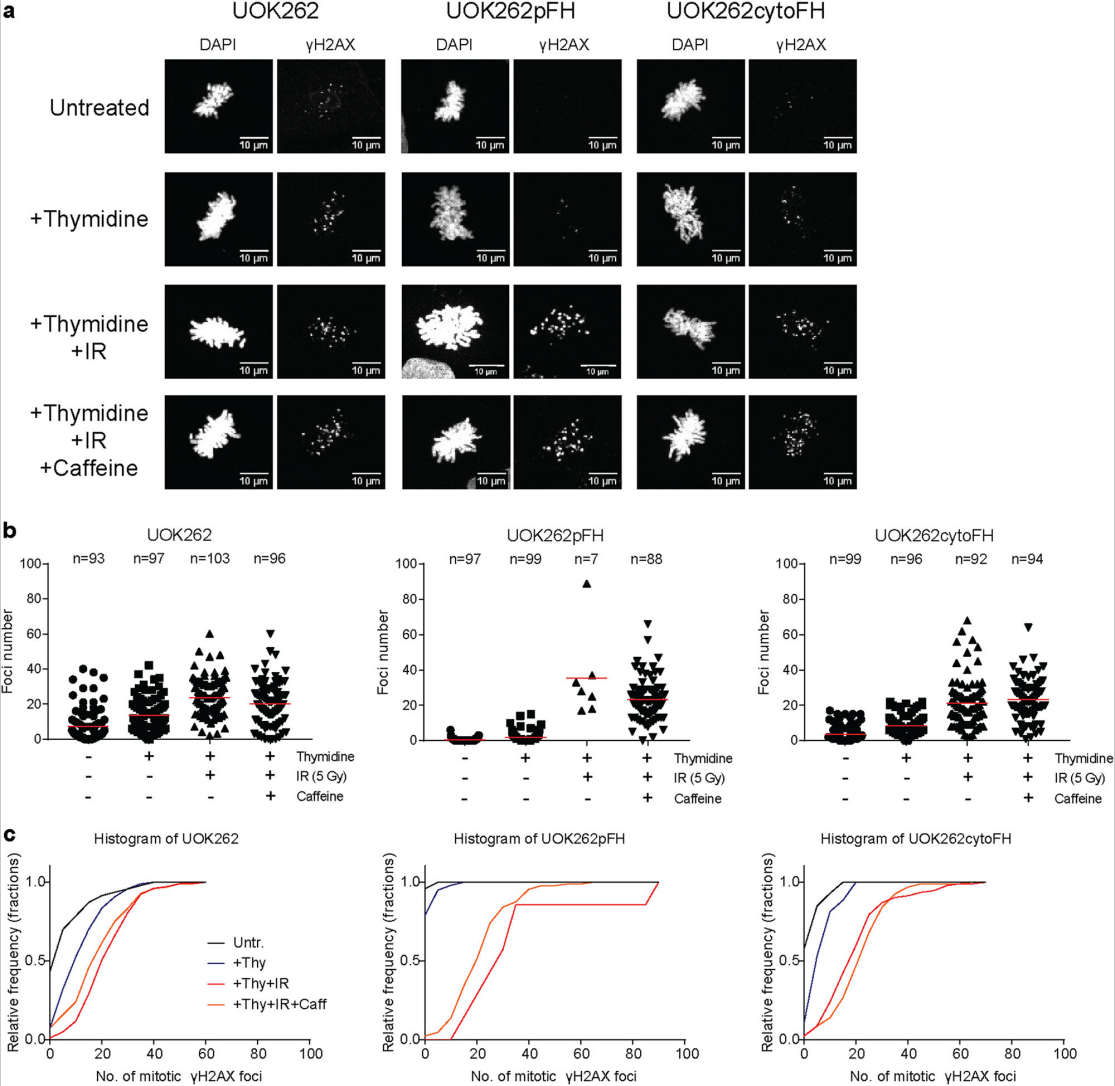
Mitotic UOK262 cells are positive for phospho-H2AX after ionising radiation (IR). **a** Representative maximal projection images of mitotic cells with the relevant treatments stained with Hoechst33342 as a nuclear marker and anti-phospho-Serine 139 H2AX (γH2AX) as a marker of DNA damage. **b**, **c** γH2AX foci count analysis using confocal microscopy from three biological replicates plotted as individual data points (**b**) or as a cumulative frequency plot (**c**). Mitotic nuclei were selected by eye based on Hoechst33342 staining showing clear condensation of chromosomes. A mitotic chromosome mask was created using ImageJ software based on Hoechst33342 staining and γH2AX foci counted using the FindFoci module. Number of mitotic cells analysed are displayed above each condition

Finally, we assessed whether UOK262 cells that enter mitosis early after the induction of damage by IR contain markers of DNA damage. To this end, we analysed γH2AX foci of thymidine-synchronised, IR-treated cells with or without caffeine treatment by confocal microscopy (Fig. 6a). As the frequency of mitotic entry had already been analysed using flow cytometry, we identified as close to 30 mitotic cells per condition per replicate as possible although this was not always possible, particularly in the IR-treated UOK262pFH cells, as MI after IR is very low. Of the mitotic cells that were analysed, we found that all cell lines exhibit mitotic nuclei with higher levels of damage after IR compared to untreated (Fig. 6b, c), suggesting that the level of persistent damage in mitosis seems to be a common feature across all cell lines. Alongside the analysis of mitotic entry by flow cytometry, these data suggest that any cell that manages to escape a checkpoint-mediated G2 arrest after IR will contain markers of DNA damage. The frequency of such an event, however, is much higher in FH-deficient cells, highlighting that fumarate accumulation may reduce the ability of G2 checkpoint signalling to prevent mitotic entry after IR. Interestingly, the number of γH2AX foci appears higher in both untreated and thymidine-synchronised UOK262 mitotic cells, suggesting these cells have a higher level of basal damage that is persisting into mitosis, consistent with the previous observation of an increase in 53BP1 foci of untreated UOK262 cells.

## Discussion

Several lines of evidence indicate that dysregulated cellular metabolism can predispose to elevated endogenous DNA damage. For instance, accelerated proliferation due to aberrant Rb/E2F signalling can induce depletion of nucleotides and elicit defects in replication^12^. Furthermore, novel links between metabolism and DNA integrity have recently been discovered, such as the translocation of ATP-citrate lyase (ACLY) in the nucleus to promote histone acetylation, facilitating homologous recombination through the recruitment of breast cancer early onset 1 (BRCA1)^13^. More recently, the mitochondrial enzyme FH was shown to translocate in the nucleus of cells subjected to DNA damage and to generate a local pool of fumarate, which, by blocking demethylation of H3K36, promotes the recruitment of proteins involved in NHEJ^9^. While the FH-fumarate-NHEJ axis appears to determine the efficiency of DNA repair in cells that express intact FH, the loss of FH observed in hereditary and sporadic cancer has recently been shown to affect the process of DNA repair^10^. Indeed, in these circumstances, FH is inactive, but fumarate accumulates, mimicking a chronic activation of the DDR. In this work, we used a well-established cell line that harbours a FH inactivating mutation and assessed the response to DNA damage induced by ionising radiation.

Our work shows that FH-deficient cells exhibit striking differences in the response to DNA damage induced by IR, when compared to an isogenic FH-reconstituted cell line. FH loss leads to resistance to ionising radiation and allows earlier cell cycle progression even in the presence of unrepaired damage after IR. Of note, we show that this response to IR is independent of mitochondrial dysfunction present in these cells and can be blunted upon expression of a cytosolic-only variant of FH, which reduces cytosolic fumarate levels but maintains defective mitochondrial function. Furthermore, the direct addition of a cell-permeable form of fumarate immediately prior to IR was sufficient to enhance G2-M transition in FH-reconstituted cell lines, suggesting the direct role of fumarate in checkpoint signalling. We also demonstrate that pro-NHEJ factors are more readily recruited and the HRR protein, Rad51, foci levels are lower in FH-deficient cells, in line with previous findings^9,10^. Interestingly, the observation that fumarate accumulation favours the error-prone NHEJ as a route of DNA repair upon damage, might explain why FH-deficient cells exhibit higher levels of DNA damage foci under basal conditions. This endogenous DNA damage could be repaired less efficiently, leading to the accumulation of mutations over time. Both the increase in basal γH2AX foci in mitosis and the increase in basal 53BP1 foci, as well as the longer duration of S phase in UOK262 cells, support the hypothesis that these cells experience increased replication stress and higher levels of endogenous damage. Interestingly, higher presence of 53BP1 nuclear bodies in G1 cells has been linked with protection of fragile sites during mitosis and higher degrees of replication stress^14,15^. Since we show that fumarate accumulation also induces a weakened G2 checkpoint, it is possible that endogenous damage is maintained via this weakened G2 checkpoint, further compounded by error-prone NHEJ and successive cell divisions, and potentiates a genomic instability phenotype. Interestingly, recent work highlighted the sensitivity of FH-deficient cell lines to the poly (ADP-ribose) polymerase (PARP) inhibitor, olaparib, known to affect cancers deficient in HRR^10^. The combination of PARP and WEE1 kinase inhibitors that abrogate G2-M checkpoint arrest has been shown to improve efficacy over olaparib alone^16,17^. Therefore, it is possible that the effect of fumarate on the G2 checkpoint, as well as the previously reported effect on HRR, could explain why FH-deficient cells appear particularly sensitive to PARP inhibition.

Besides this specific role of fumarate in NHEJ, many known features of FH loss could explain an increase in endogenous damage and the level of fumarate accumulation positively correlates with the level of endogenous damage^10^. FH loss leads to the accumulation of reactive oxygen species, which are known to induce DNA damage^18^. Furthermore, a potential nucleotide imbalance, known to lead to genomic instability and replication stress^12^, could be caused by impairment of both de novo pyrimidine and purine biosynthesis due to mitochondrial dysfunction and product inhibition of phosphoribosylaminoimidazolesuccinocarboxamide (SAICAR) lyase in the pentose phosphate shunt, respectively^19^. Fumarate can also bind cysteine residues in a process termed succination^20^. It is currently unclear as to how many processes are affected by succination in FH-deficient cancers but an investigation into the potential role of succination in DNA damage checkpoint signalling would be worth exploring. In addition to its effects on metabolism, fumarate is a well-documented epigenetic modifier. For instance, we recently showed that fumarate elicits an epithelial-to-mesenchymal transition via inhibition of DNA cytosine demethylation and subsequent epigenetic suppression of *miR200*^4^. Interestingly, changes to chromatin structure via loss of bromodomain protein 4 (Brd4) have been shown to facilitate DNA repair and promote checkpoint recovery^21^. It is, therefore, possible that that the aberrant epigenetic landscape in FH-deficient cells could also contribute to defects in DDR and to overcome an IR-induced checkpoint. Indeed, as the cell lines used in this study share the same genetic background, the phenotypes observed here likely represent those that are reversibly induced by fumarate accumulation consistent with an epigenetic-associated mechanism. However, with the evidence that even short-term addition of fumarate can enhance G2-M transition, any potential epigenetic changes must occur over a short time-frame and future work should aim to explore these mechanisms further.

In conclusion, these data highlight the importance of fumarate accumulation in the context of the DDR and provide a potential model for how fumarate accumulation promotes genomic alterations that could give rise to cancer formation in HLRCC patients. It would be important to assess the extent of DNA mutations in these patients and identify possible mutations that cooperate with FH during tumorigenesis.

## Material and methods

### Cell lines

UOK262, UOK262pFH and UOK262cytoFH-V5 cell lines were previously characterised^4,22^. The UOK262cytoFH cell line was generated by expressing the FH cDNA sequence lacking the mitochondrial translocation sequence (see cloning section for details). All cell lines were cultured in DMEM high glucose with pyruvate (Gibco™, Cat. No. 11995073) supplemented with 10% fetal bovine serum (FBS) (Gibco™, Cat. No. 26140079). All cells were cultured at 37 °C and 5% CO_2_.

### Ionising radiation experiments

All experiments involving irradiation used the Xstrahl at 195 kV and 10 mA following the manufacturer’s instructions to achieve the desired level of irradiation in Grays. For addition of exogenous fumarate, 400 µM monomethyl-fumarate (Sigma) was added to the cell culture media immediately prior to irradiation.

### Protein extraction and western blotting

Cell lysates were prepared using RIPA buffer supplemented with phosphatase inhibitor cocktails 2 & 3, protease inhibitor cocktail, Benzonase® Nuclease (All Sigma) and 4.5 mM MgCl_2_ used as per the manufacturer’s instructions. Protein was quantified using a BCA kit (Pierce) and 50 µg protein plus Sample Buffer was run on a Bolt® 4–12% Bis-Tris pre-cast gel alongside a Novex® Sharp Prestained Protein Standard in MES running buffer, before transfer to nitrocellulose membrane using the iBlot® semi-dry transfer system all according to manufacturer’s instructions (All Life Technologies). The membranes were stained using Ponceau S to determine equal loading and subsequently blocked using 5% (w/v) milk (Marvel) in TBS-T (1 x phosphate-buffered saline (PBS), 0.1% Tween-20) for 1 h. Membranes were placed in 2 ml of the primary antibody in blocking solution using 50 ml Falcon tubes and rotated overnight at 4 °C. Membranes were incubated with secondary antibody (Licor) in blocking solution for 1 h at room temperature. The visualisation of proteins was performed using the Odyssey® Infrared Imaging system (Licor). The primary/secondary antibodies and their respective dilutions were as follows:

V5 mouse 1:2000 Life Technologies R960–25

FH Clone 2A2 mouse 1:500 Origene TA500681

Calnexin Rabbit 1:1000 Abcam Ab22595

Phospho-Serine 139 H2AX (Clone JBW301) mouse 1:500 Merck Millipore 05–636

GFP rabbit 1:2000 Abcam Ab290

β-Actin rabbit 1:1000 Cell Signaling 4967S

IR-Dye 680RD goat anti-mouse 1:2000 LI-COR 925–68070

IR-Dye 680RD goat anti-rabbit 1:2000 LI-COR 925–68071

IR-Dye 800CW goat anti-mouse 1:2000 LI-COR 925–32210

IR-Dye 800CW goat anti-rabbit 1:2000 LI-COR 925–32211

### SRB colorimetric assay

Cells were seeded in six replicate wells at 1000 cells/well in a Nunc™ MicroWell™ 96-Well Microplate in replicate plates with 1, 3, 5 or 10 Gy of irradiation being performed approximately 8 h after seeding. After 5 days, cell media was removed and wells washed once with 1xPBS. All plates were then fixed in ice-cold 1% TCA for 30 min at 4 °C, then washed with tap water three times before being air-dried overnight. Once dry, 100 µl of 0.057% (wt/vol) SRB solution was added to each well and incubated at room temperature for 30 min then washed three times with 1% acetic acid before being air-dried overnight. Once dry, the bound SRB stain was dissolved in 100 µl of 10 mM Tris (pH 8.0). Absorbance at 560 nm was measured using Tecan Infinite® M200 plate reader.

### Colony formation assay

Cells were seeded at 200,000 cells/well in a Nunc™ 60 mm^2^ dish in replicate dishes then irradiated 48 h later at 1, 3, 5 or 10 Gy. Twenty-four hours post-irradiation, cells were then seeded in a Nunc™ 6-well plate at 200 cells/well for the UOK262 and 100 cells/well for the UOK262pFH to account for differences in plating efficiency. The remaining cells were processed for DNA content analysis. Cells were then cultured for 2 weeks with the media being changed after 1 week. Colonies were fixed and stained as per the SRB protocol and counted.

### DNA content analysis for flow cytometry

Cells were fixed with 1 mL ice-cold 70% ethanol overnight, washed and re-suspended in 1xPBS containing 2% bovine serum albumin (BSA), 0.1% Tween-20 and 0.1% Triton X-100 (blocking buffer) and incubated at room temperature for 1 h. Cells were then incubated at room temperature for 2 h in blocking solution containing primary antibody. Cell were washed three times in blocking buffer and re-suspended in blocking buffer containing the appropriate secondary antibody and incubated for 1 h at room temperature in the dark. DNA was stained using either 40 μg/ml Propidium iodide (PI, Thermo Fisher, Cat. No. GTX70230) with 100 µg/ml RNAse A or 1 µg/ml FxCycle™ Violet (Thermo Fisher, Cat. No. F10347) following the manufacturer’s instructions. The BD Biosciences LSRFortessa™ flow cytometer was used for processing samples with the appropriate laser/detectors. Analysis of recorded results was performed using the FlowJo® V9 software. Dyes/Antibodies used for flow cytometry were:

PI-40 µg/ml Thermo Fisher GTX70230

FxCycle Violet-1 µg/ml Thermo Fisher ab 133534

Phospho-MPM2 mouse 1:500 Merck Millipore 05–368--

Alexa Flour 488 Goat anti-mouse 1:500 Thermo Fisher A11017

Alexa Flour 568 Goat anti-mouse 1:500 Thermo Fisher A11019532

### Immunofluorescence

Cells were fixed with either 4% formaldehyde for 10 min or ice-cold 100% methanol for 5 min at room temperature. Cells were permeabilised with 0.1% Triton X-100, 0.1% Tween-20, 1xPBS (PBSTT) for 10 min at room temperature and then incubated with primary antibody in blocking solution, PBSTT with 2% BSA, for 1 h at room temperature (except for phospho-DNA-PKcs which was incubated overnight). Samples were washed with PBSTT three times and then incubated in blocking solution containing secondary for 1 h at room temperature. Samples were washed once with PBSTT and a further two times with 1xPBS and placed at 4 °C in the dark. Coverslips were mounted with Vectashield containing DAPI (Vector Laboratories, Cat. No H-1200) and subsequently sealed with nail polish. Ninety-six-well plates were sealed with plastic film (VWR, Cat. No. 731–0319). Antibodies, dilutions and which fixative was used were as follows:

RAD51 rabbit 1:2000 Abcam ab133534 PFA

Phospho-Serine 139 H2AX (Clone JBW301) mouse 1:2000 Merck Millipore #05–636 PFA

pH3 mouse 1:2000 abcam ab14955 PFA

53BP1 rabbit 1:1000 Novus Biologicals NB 100–304 PFA

Phospho-Serine 2056 DNA-PKcs rabbit 1:450 Abcam ab18192 100% Methanol

TOMM20 rabbit 1:500 Abcam ab186735 PFA

Alexa Flour 488 Goat anti-mouse 1:500 Thermo Fisher A11017-

Alexa Flour 488 Goat anti-rabbit 1:500 Thermo Fisher A11034-

Alexa Flour 568 Goat anti-mouse 1:500 Thermo Fisher A11019-

Alexa Flour 568 Goat anti-rabbit 1:500 Thermo Fisher A11036-

### Cell synchronisation and the G2-M transition

Cells were treated with or without 2 mM thymidine (Sigma-Aldrich; Cat. No. T1895–5G) for 16 h, followed by 8 h release and a second treatment of 2 mM thymidine for 16 h. Cells were then released into fresh media and harvested at different time points for cell cycle analysis. Subsequent maximal G2 content was identified as 9 h after second thymidine release for UOK262 and UOK262cytoFH cells and 7 h after release for UOK262pFH cells. Induction of DSBs by IR was performed at these time points where G2 was maximal. Seven hours after IR treatment, 200 μg/ml nocodazole (Sigma-Aldrich; Cat. No. M1404-2MG) with or without addition of 4 mM caffeine (Sigma-Aldrich; C0750) was added to the relevant dishes in order to enrich for mitotic cells and inhibit ATM/ATR signalling, respectively.

### Metabolite extraction and analysis

Cells were plated in triplicate plus an extra well for cell counting and cultured in standard medium for 18 h. Metabolite extraction buffer (MEB) was prepared composing of 50% methanol and 30% acetonitrile in water and kept at 4 °C. The counting well was then counted using the Invitrogen™ Countess™ automated cell counter based on manufacturer’s instructions to give the total number of cells per well. The media from the replicate wells was removed and the cells were washed once with 1xPBS. Metabolites were then extracted by adding 1 mL MEB per million cells and quickly scraped. The insoluble material was immediately pelleted in a cooled centrifuge (4 °C) at 13,000 rpm for 15 min and the supernatant was collected for subsequent LC–MS analysis. A HILIC column (4.6 × 150 mm, guard column 2.1 × 20 mm, Merck) was used for LC separation. The aqueous mobile phase solvent used was 0.1% formic acid in water (solvent A) and the organic mobile phase was 0.1% formic acid in acetonitrile (solvent B). The flow rate was set at 300 μl/min and the column oven set to 30 °C. The mobile phase gradient was described previously^4^. Subsequent analysis was performed using the Xcalibur Quan Browser software (Thermo Fisher Scientific).

### Oxygen consumption rate (OCR) measurements

Oxygen consumption rate (OCR) was measured using the real-time flux analyser XF-24e (Seahorse Bioscience) as previously described (ref). In brief, 4 × 104 cells were seeded and left untreated overnight, and then treated with 1 μM oligomycin, 2 μM carbonyl cyanide-p-trifluoromethoxyphenylhydrazone (CCCP), rotenone and antimycin A (both 1 μM) (all Sigma-Aldrich). In the case of later experiments, OCR was normalised to protein content where, after the experiment, cells were lysed using RIPA buffer containing protease inhibitors (25 mM Tris/HCl pH 7.6, 150 mM NaCl, 1% NP-40, 1% sodium deoxycholate, 0.1% SDS). The protein content of each well was measured using a BCA kit (Pierce) following the manufacturer’s instructions. In the case of normalisation, OCR is expressed as pmol/min/µg protein.

### Cloning

To generate the GFP-tagged plasmids, the original pEF1α-V5/His construct was modified using a “restriction-free” cloning method (see www.rf-cloning.org). This approach uses two sequential PCR reactions followed by DpnI-mediated digestion of the original plasmid. Briefly, primers were designed that share 50% homology with the intended insert sequence (in this case AcGFP sequence) and 50% homology with the sites of insertion within the backbone vector. Primers sequence is indicated below. The PCR was performed using the Phusion DNA polymerase and primers at a final concentration of 0.5 µM with the pAcGFP-N1 vector as a template to copy the GFP sequence. The PCR conditions are set out below:

Denature 98 °C 30 s 1X

Denature 98 °C 8 s

Anneal 57–60 °C 20 s 35X

Extension 72 °C 15 s

Final Extension 72 °C 5 min 1X

The second PCR was performed using 120 ng DNA generated in the first PCR reaction and 70 ng of the pEF1α-V5/His vector as the destination vector using the following PCR conditions:

Denature 98 °C 30 s 1X

Denature 98 °C 8 s

Extension 72°2 min 15 s 15X

Final extension 72 °C 5 min 1X

Competent cells were transformed with 1 µl of the final PCR product using One Shot® MAX Efficiency® DH5α™-T1R chemically-competent cells (Invitrogen) and single colonies grown for plasmid DNA extraction. Sequencing of individual plasmids confirmed the replacement of the V5 tag with GFP sequence and the generated pEF1α-GFP plasmid. Primers were then designed to recognise FH cDNA that does not contain the endogenous mitochondrial targeting sequence (MTS) and included the *Spe*I and *Eco*RI restriction sites alongside a buffer sequence of GATC. PCR with these primers Phusion DNA polymerase using pEF1α-FH-V5 vector as a template yielded a band of the correct size (~1.5 kb) and this purified as stated above. The purified FH PCR product and the recently generated pEF1α-GFP vector were then subject to digestion by *Spe*I (NEB, Cat. No. R3133S) and *Eco*RI (NEB, Cat. No. R3101S) HF restriction enzymes. The pEF1α-GFP backbone and FH insert were purified, ligated and used to transform bacterial competent cells as described above. Sequencing confirmed the presence of the FH insert, to generate the cytoFH-GFP plasmid. The vector was transfected into cells seeded into a 6-well plate using Lipofectamine 2000 (Thermo Fisher, Cat. No 11668027) using 2.5 µg plasmid DNA and 9 µl Lipofectamine 2000 reagent per well. Cells positive for GFP were sorted as a mixed, medium-expressing population using a BD Influx FACS machine.

Primer Sequence Restriction sites

FH_R2 GATCGAATTCCTTTGGACCCAGCATGTCCTTAGG *Eco*RI

FH_F4 GATCGGTACCATGGCAAGCCAAAATTCCTTCCGG *Kpn*I

RF_F1 CGAGTCTAGAGGGCCCTTCGAAATGGTGAGCAAGGGCGCC Restriction free

RF_R1 GGCTGATCAGCGGGTTTAAACTCACTTGTACAGCTCATCCATGCC Restriction free

### Statistical analysis

All pair-wise analysis of statistical significance was determined using multiple unpaired *t*-tests using the Holm-Sidak multiple comparison correction. For any test using multiple comparisons between multiple groups (Fig. 5b–d) a Tukey’s multiple comparison test was used. For single comparisons only, a Mann–Whitney *U*-test was applied (Fig. 2f). Only the adjusted *p*-values of relevant comparisons that are lower than or close to 0.05 have been added to the final figures, non-significant comparisons were not displayed. Data presented as mean ± SD for any flow cytometry data, mean ± SEM for all other graphs and contained at least three biological replicates.

## Electronic supplementary material

Supplementary Figure 1

Supplementary Figure 2

Supplementary Figure 3

Supplementary Figure 4

Supplementary Figure 5

Supplementary Figure 6

Supplementary Figure Legends
