# Supplementary figures and images for: Fumarate hydratase loss promotes mitotic entry in the presence of DNA damage after ionising radiation
Source: Cell Death Dis. 2018 Sep 6;9(9):913. doi: 10.1038/s41419-018-0912-3 (PMC6127199; doi:10.1038/s41419-018-0912-3)

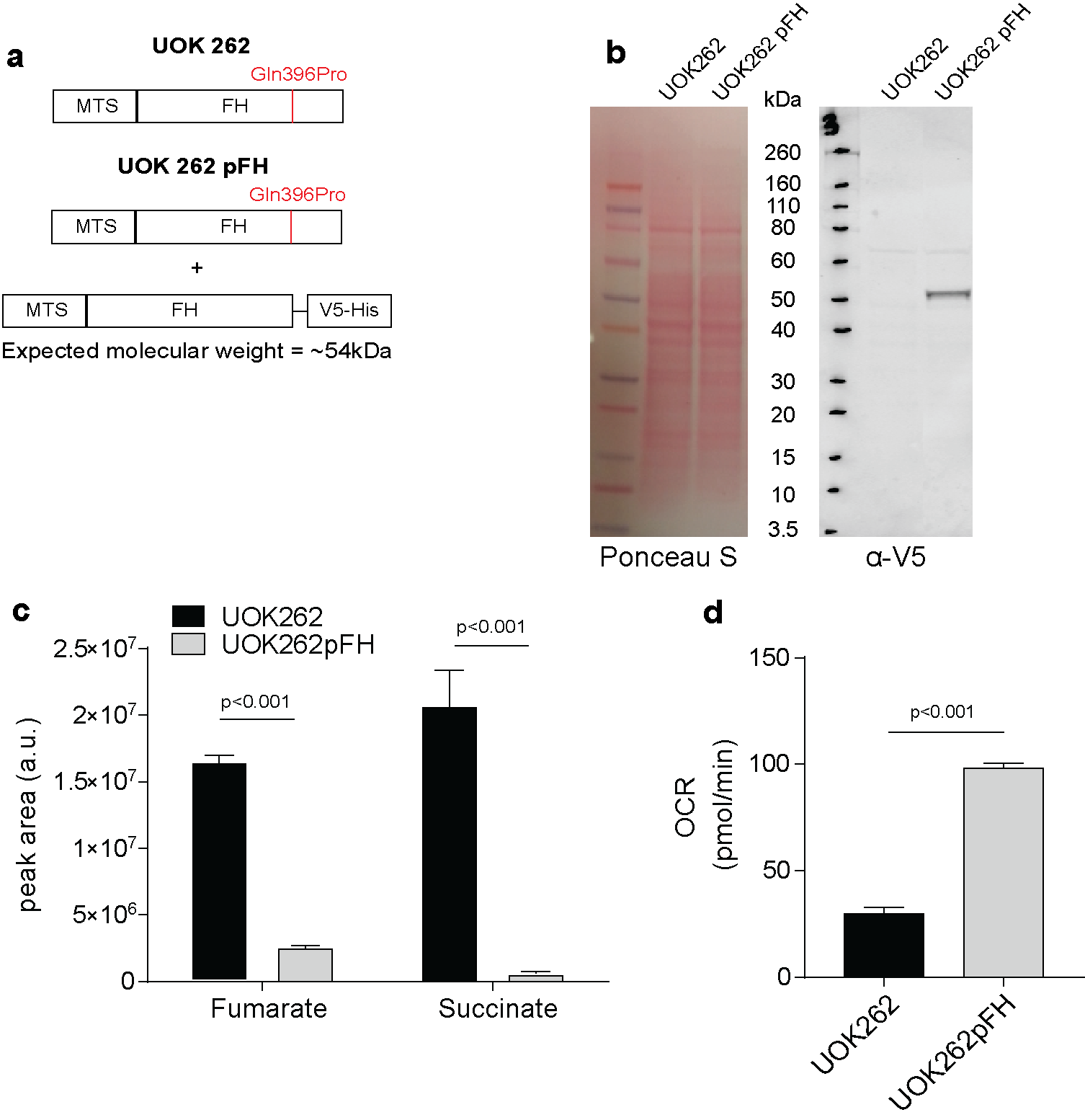

Supplement: Supplementary file 1 — Supplementary Figure 1 [file 41419_2018_912_MOESM1_ESM.tif]

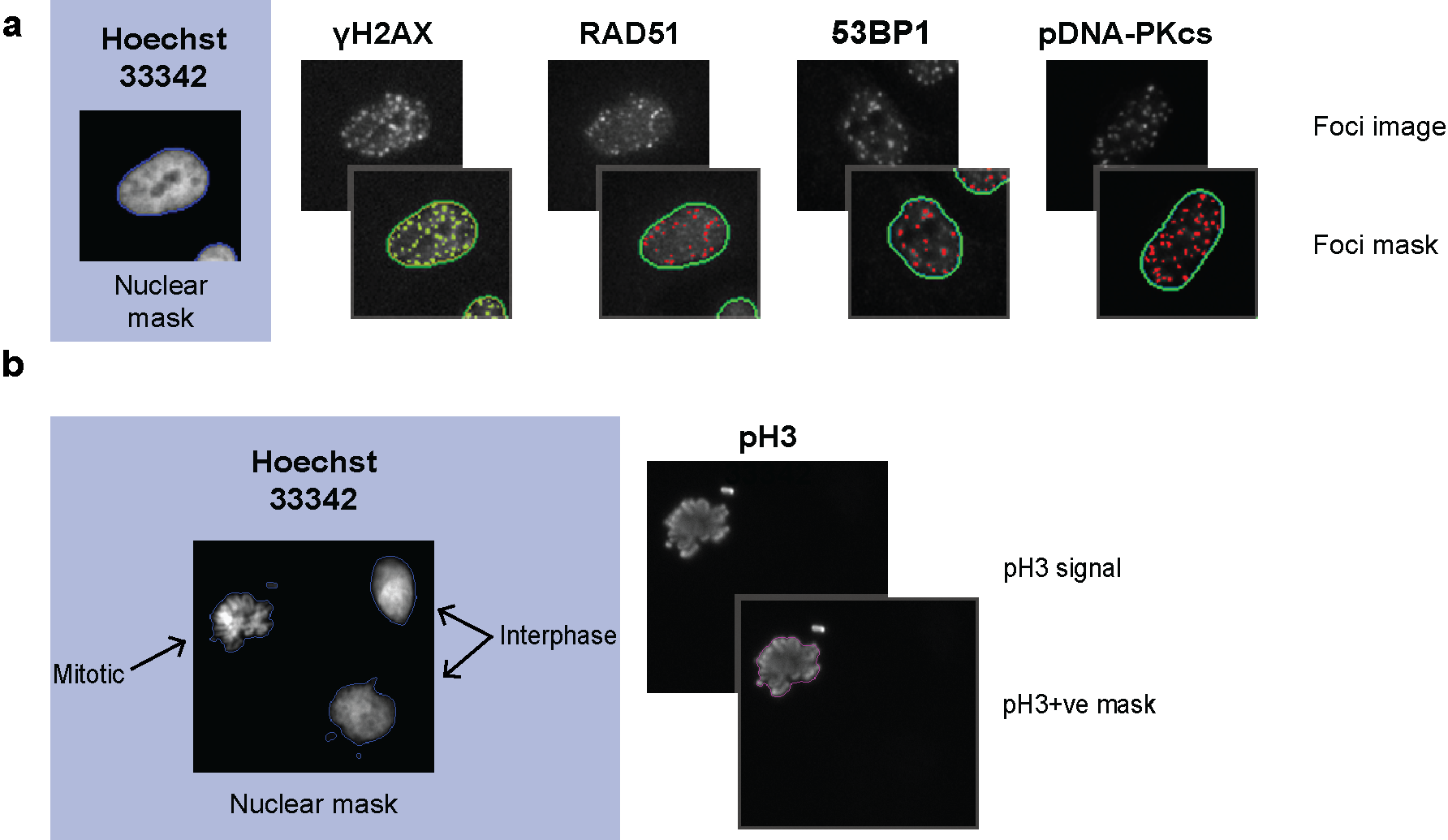

Supplement: Supplementary file 2 — Supplementary Figure 2 [file 41419_2018_912_MOESM2_ESM.tif]

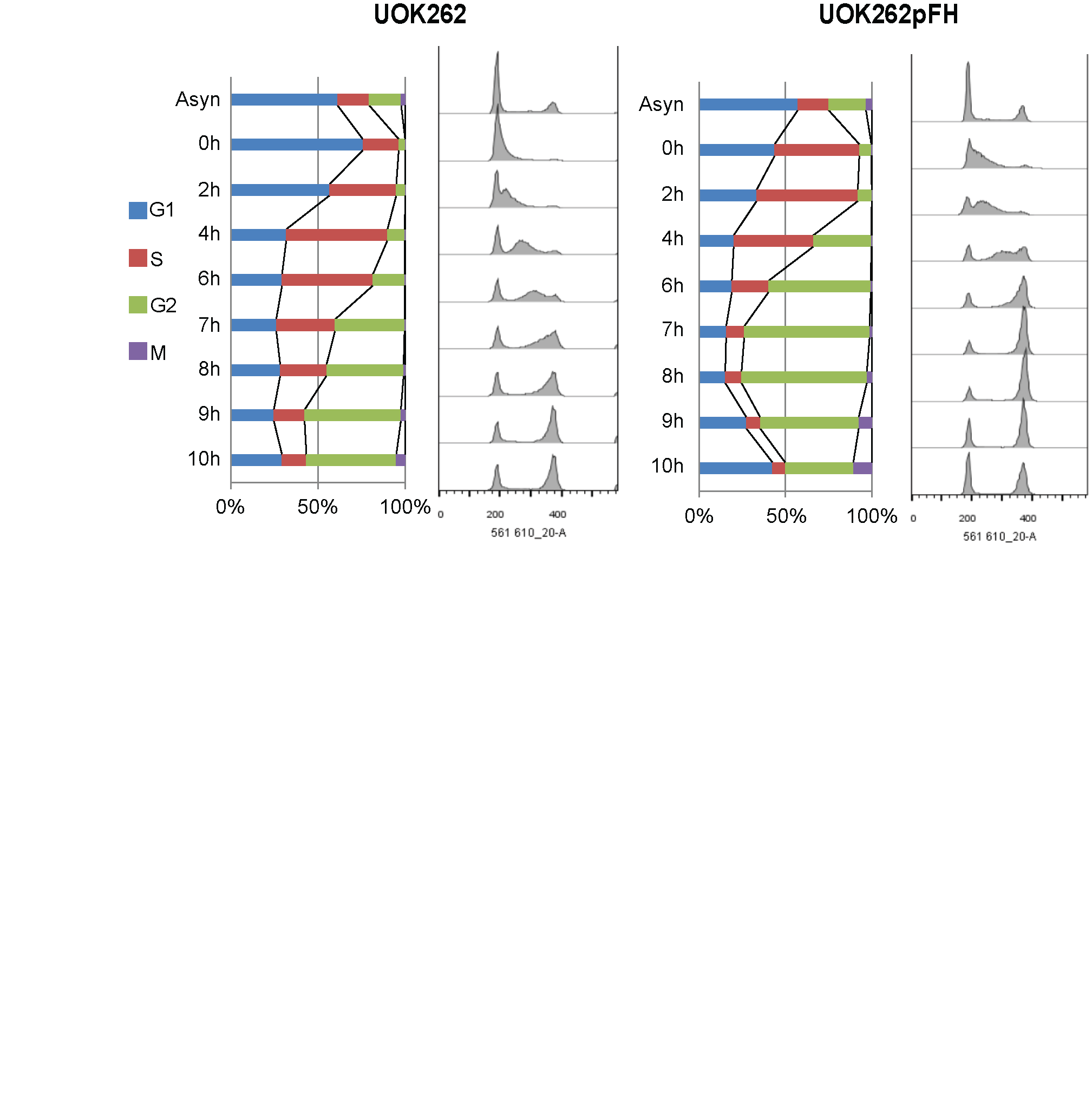

Supplement: Supplementary file 3 — Supplementary Figure 3 [file 41419_2018_912_MOESM3_ESM.tif]

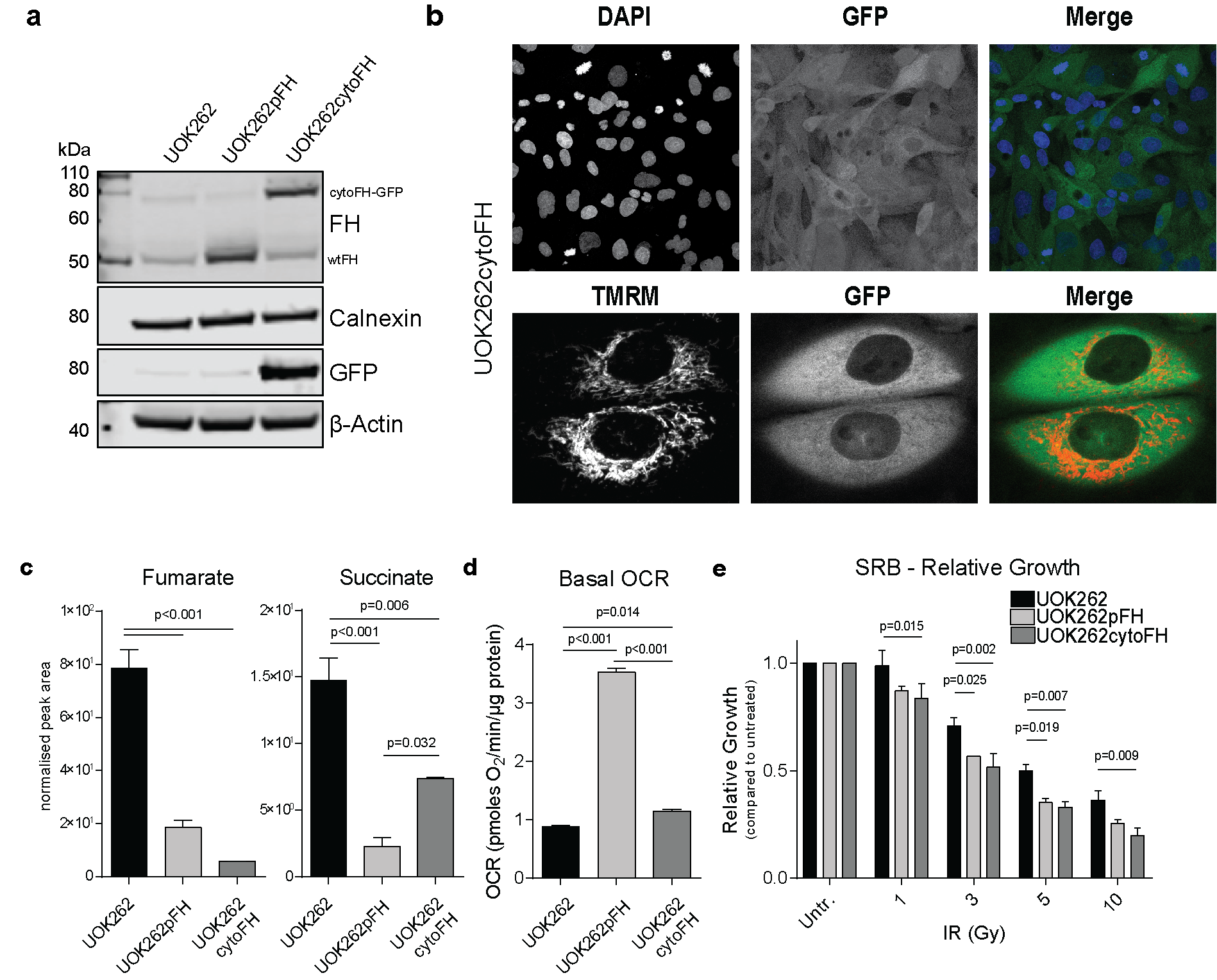

Supplement: Supplementary file 4 — Supplementary Figure 4 [file 41419_2018_912_MOESM4_ESM.tif]

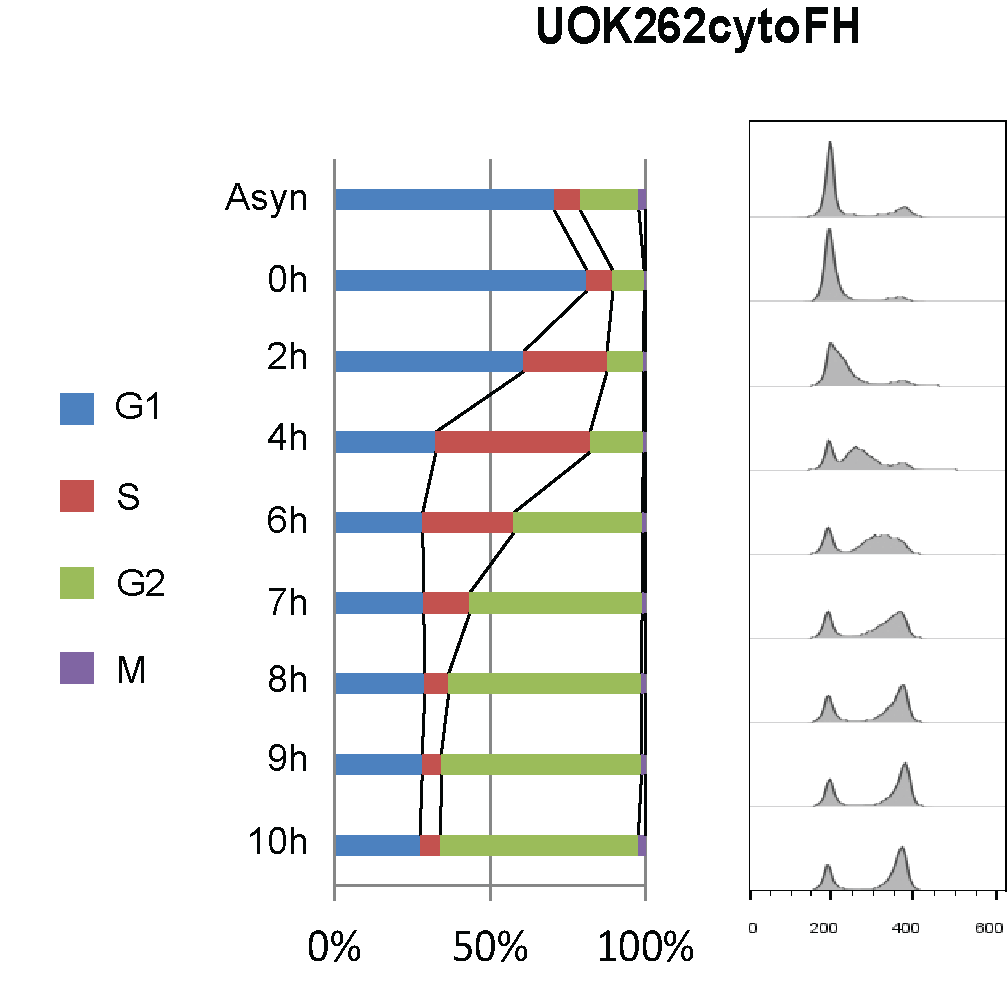

Supplement: Supplementary file 5 — Supplementary Figure 5 [file 41419_2018_912_MOESM5_ESM.tif]

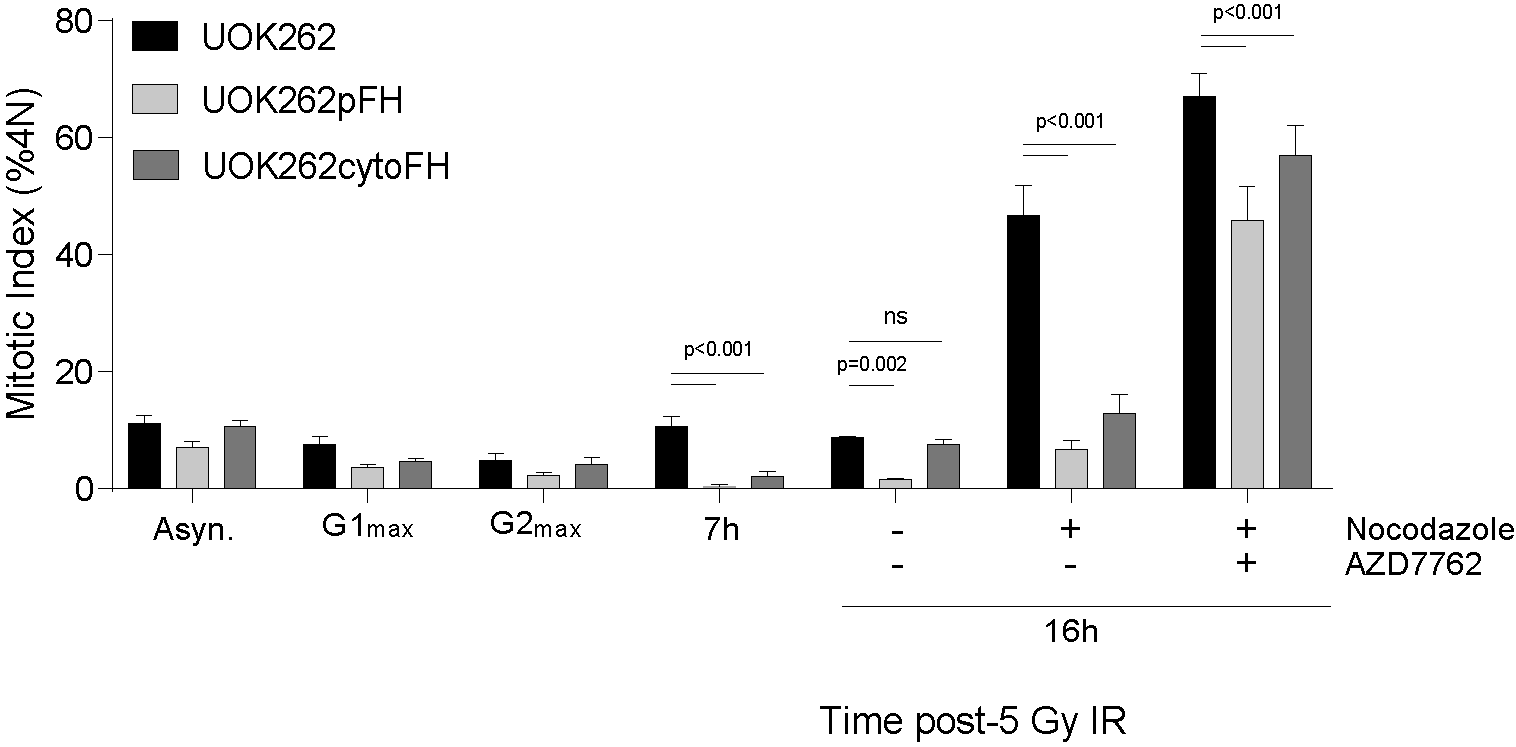

Supplement: Supplementary file 6 — Supplementary Figure 6 [file 41419_2018_912_MOESM6_ESM.tif]
