# Supplementary material for: Fumarate hydratase loss promotes mitotic entry in the presence of DNA damage after ionising radiation
Source: Cell Death Dis. 2018 Sep 6;9(9):913. doi: 10.1038/s41419-018-0912-3 (PMC6127199; doi:10.1038/s41419-018-0912-3)
Supplement: Supplementary file 7 — Supplementary Figure Legends [file 41419_2018_912_MOESM7_ESM.docx]

**SUPPLEMENTARY FIGURES LEGENDS**

**Figure S1: FH re-expression rescues the metabolic phenotypes present in UOK262 cells.** a. Schematic showing the status of FH in UOK262 and UOK262pFH cell lines. MTS, mitochondrial targeting sequence b. Western blot analysis using anti-V5 antibodies and Ponceau S protein stain as a loading control. c. Intracellular abundance of fumarate and succinate after metabolite extraction and subsequent analysis by LC-MS. Adjusted p-values calculated using multiple unpaired t-tests using the Holm-Sidak multiple comparison correction. d. Basal oxygen consumption rate (OCR) measurements using the Seahorse XFe 24 analyser. P-values calculated using a paired, two-tailed parametric t-test.

**Figure S2: Mask generation for analysis of high content microscopy.** a. Representative images of nuclear and foci masks created using the Arrayscan software. b. Representative images of nuclear and phospho-Serine 10 Histone H3 (pH3) masks created using the Arrayscan software.

**Figure S3: Synchronisation of cells by a double thymidine block.** UOK262 and UOK262pFH cells were synchronised using a double thymidine block and cells were harvested for cell cycle analysis at sequential time points after the second thymidine release. Cell cycle was determined by DNA content and mitotic index (MI) using propidium iodide (PI) and phospho-mpm-2 staining (p-mpm2). Cell cycle phases were plotted as percent (left panel) alongside individual plots of DNA content (right panel).

**Figure S4: Characterisation of UOK262cytoFH cells.** a. Western blot analysis of protein extracts from UOK262, UOK262pFH and UOK262cytoFH cell lines using anti-FH and anti-GFP antibodies with anti-β-Actin and anti-Calnexin antibodies as loading controls. b. Representative confocal images of UOK262wtFH-GFP cells stained with DAPI, TMRM and residual GFP fluorescence from the FH fusion protein. c. Intracellular levels of fumarate and succinate analysed by LC-MS in the indicated cell lines. Peak area was normalised to HEPES, used as internal standard in the extraction solution. Adjusted p-values calculated using a One-way ANOVA with Tukey’s multiple comparisons test d. Basal Oxygen consumption rate (OCR) averaged across the first three measurements from three biological replicates measured across the indicated cell lines. Adjusted p-values calculated using a One-way ANOVA with Tukey’s multiple comparisons test e. Relative growth as compared to untreated control using Sulforhodamine B (SRB) absorbance at 564 nm after increasing exposure to ionising radiation (IR). Adjusted p-values calculated using a Two-way ANOVA with a Dunnett’s multiple comparison test using UOK262 cells as a control group.

**Figure S5: Synchronisation of UOK262cytoFH cells by a double thymidine block.** UOK262cytoFH cells were synchronised using a double thymidine block and cells were harvested for cell cycle analysis at sequential time points after the second thymidine release. Cell cycle was determined by DNA content and mitotic index using propidium iodide (PI) and phospho-mpm-2 staining (p-mpm2). Cell cycle phases were plotted as percent (left panel) alongside individual plots of DNA content (right panel).

**Figure S6: Inhibition of Checkpoint kinase (Chk) 1/2 by AZD7762 induces mitotic entry in UOK262pFH and UOK262cytoFH cells after IR.** UOK262 and UOK262pFH cells were synchronised using a double thymidine block and irradiated with 5 Gy ionising radiation (IR) at G2_max_. Nocodazole was added with or without the Chk1/2 inhibitor AZD7762 to the relevant cells 7 hours post IR. Cells were harvested and fixed at the relevant times and cell cycle dynamics determined by DNA content and mitotic index using the DNA stain FXCycle Violet and phospho-mpm-2 staining (p-mpm2). Mitotic p-mpm2-+ve cells were then expressed as a fraction of the 4N population. Adjusted p-values calculated using a Two-way ANOVA with a Dunnett’s multiple comparisons test using UOK262 cells as a control group.
